# Hidden risk in normal myocardial perfusion scans: AI-detected proximal coronary calcium on CT attenuation maps improves prognosis

**DOI:** 10.1007/s00259-026-08084-x

**Published:** 2026-07-28

**Authors:** Jianhang Zhou, Robert J.H. Miller, Aakash Shanbhag, Aditya Killekar, Donghee Han, Krishna K. Patel, Konrad Pieszko, Jirong Yi, Meghana Kiran Urs, Giselle Ramirez, Mark Lemley, Paul B. Kavanagh, Joanna X. Liang, Assiata Kamagate, Valerie Builoff, Andrew J. Einstein, Attila Feher, Edward J. Miller, Albert J. Sinusas, Terrence D. Ruddy, Stacey Knight, Viet T Le, Steve Mason, Panithaya Chareonthaitawee, Samuel Wopperer, Erick Alexanderson, Isabel Carvajal-Juarez, Thomas L. Rosamond, Leandro Slipczuk, Mark I. Travin, René R. S. Packard, Wanda Acampa, Mouaz Al-Mallah, Robert A. deKemp, Ronny R. Buechel, Daniel S. Berman, Damini Dey, Marcelo F. Di Carli, Piotr J. Slomka

**Affiliations:** 1 Departments of Medicine (Division of Artificial Intelligence in Medicine), Biomedical Sciences, and Cardiology, Cedars-Sinai Medical Center, 6500 Wilshire Blvd, Los Angeles, CA 90048 USA; 2 Department of Cardiac Sciences, University of Calgary, Calgary, AB Canada; 3 Signal and Image Processing Institute, Ming Hsieh Department of Electrical and Computer Engineering, University of Southern California, Los Angeles, CA USA; 4 Departments of Medicine (Cardiology) and Population Health Science and Policy, Icahn School of Medicine at Mount Sinai, New York, NY USA; 5 Department of Interventional Cardiology and Cardiac Surgery, University of Zielona Góra, Zielona Góra, Poland; 6 Division of Cardiology, Department of Medicine, Department of Radiology, Columbia University Irving Medical Center and New York- Presbyterian Hospital, New York, NY USA; 7 Section of Cardiovascular Medicine, Department of Internal Medicine, Yale University School of Medicine, New Haven, CT USA; 8 Division of Cardiology, University of Ottawa Heart Institute, Ottawa, ON Canada; 9 Intermountain Medical Center Heart Institute, Intermountain Healthcare, Murray, UT USA; 10 Department of Internal Medicine, University of Utah, Salt Lake City, UT USA; 11 Department of Cardiovascular Medicine, Mayo Clinic, Rochester, MN USA; 12 Department of Nuclear Cardiology, National Institute of Cardiology Ignacio Chávez, México City, México; 13 Faculty of Medicine, National Autonomous University of Mexico, México City, México; 14 Department of Cardiovascular Medicine, The University of Kansas Medical Center, Kansas City, KS USA; 15 Cardiology Division, Montefiore Health System/Albert Einstein College of Medicine, Bronx, NY USA; 16 Department of Radiology (Nuclear Medicine), Montefiore Medical Center, Albert Einstein College of Medicine, Bronx, NY USA; 17 Division of Cardiology, Department of Medicine, David Geffen School of Medicine, University of California, Los Angeles, CA USA; 18 Department of Advanced Biomedical Sciences, University of Naples Federico II, Naples, Campania Italy; 19 Houston Methodist DeBakey Heart & Vascular Center, Houston Methodist Academic Institute, Houston, TX USA; 20 Division of Cardiology, Department of Medicine, University of Ottawa Heart Institute, Ottawa, ON Canada; 21 Department of Nuclear Medicine, Cardiac Imaging, University Hospital Zurich, Zurich, Switzerland; 22 Cardiovascular Imaging Program, Departments of Radiology and Medicine; Division of Nuclear Medicine and Molecular Imaging, Department of Radiology; and Cardiovascular Division, Department of Medicine, Brigham and Women’s Hospital, Boston, MA USA

**Keywords:** Proximal coronary artery calcium, Artificial intelligence, Myocardial perfusion imaging, Computed tomography, Major adverse cardiovascular events, All-cause mortality

## Abstract

**Purpose:**

Spatial distribution of coronary artery calcium (CAC) may provide additional prognostic value in patients undergoing SPECT and PET myocardial perfusion imaging (MPI). We aimed to automatically identify CAC in proximal segments from attenuation correction CT (CTAC) scans using artificial intelligence (AI) and to evaluate prognostic significance in two large international multicenter registries.

**Methods:**

From hybrid MPI/CT imaging (*N* = 43,099) across 15 sites, we included 4,552 most relevant patients with (1) no prior coronary artery disease; (2) AI-derived mild CAC scores (1–99); and (3) normal perfusion (stress total perfusion deficit < 5%). The independent associations between AI-identified proximal CAC and major adverse cardiovascular events (MACE) and all-cause mortality (ACM) were evaluated using multivariable Cox regression, likelihood ratio test (LRT), and continuous net reclassification index (NRI).

**Results:**

Among the patients with mild CAC and normal perfusion (mean age 65 ± 12 years, 51% male), 1,730 (38%) had proximal CAC. Over 3.6 (inter-quartile interval 2.1, 5.2) years follow-up, 599 (13%) and 444 (10%) patients had MACE or ACM, respectively. Proximal CAC was associated with an increased risk of MACE (adjusted hazard ratio [HR] 1.24, 95% CI 1.03–1.48, *P* = 0.02) and ACM (adjusted HR 1.25, 95% CI 1.01–1.53, *P* = 0.04) after the adjustment of CAC score and density, clinical risk factors, and perfusion deficit. Proximal CAC improved the risk stratification of MACE (LRT *P* = 0.02; NRI 12%) and ACM (LRT *P* = 0.04; NRI 12%).

**Conclusion:**

In patients with mild CAC and normal myocardial perfusion, AI-based proximal CAC detection identified a subgroup at increased risk of adverse outcomes. Automated identification of proximal CAC may provide incremental prognostic information beyond perfusion findings and CAC scoring and could improve risk stratification in patients who might otherwise be considered low risk.

**Graphical abstract:**

From patients who underwent hybrid myocardial perfusion imaging (MPI) from 15 sites, we analyzed those without prior coronary artery disease (CAD), mild coronary artery calcium (CAC) scores (1-99), and normal perfusion (stress total perfusion deficit <5%). A previously developed AI model was used to identify CAC lesions in proximal coronary segments on CT attenuation correction maps (CTAC). We evaluated associations with major adverse cardiovascular events (MACE) and all-cause mortality (ACM), showing risk stratification of proximal CAC and improvement by net reclassification index (NRI). CAC lesion color: green, left anterior descending artery (LAD) with left main artery; red, left circumflex artery (LCX); yellow, right coronary artery (RCA). Adjusted hazard ratios (HRs) are shown with 95% confidence intervals.

**Figure:**
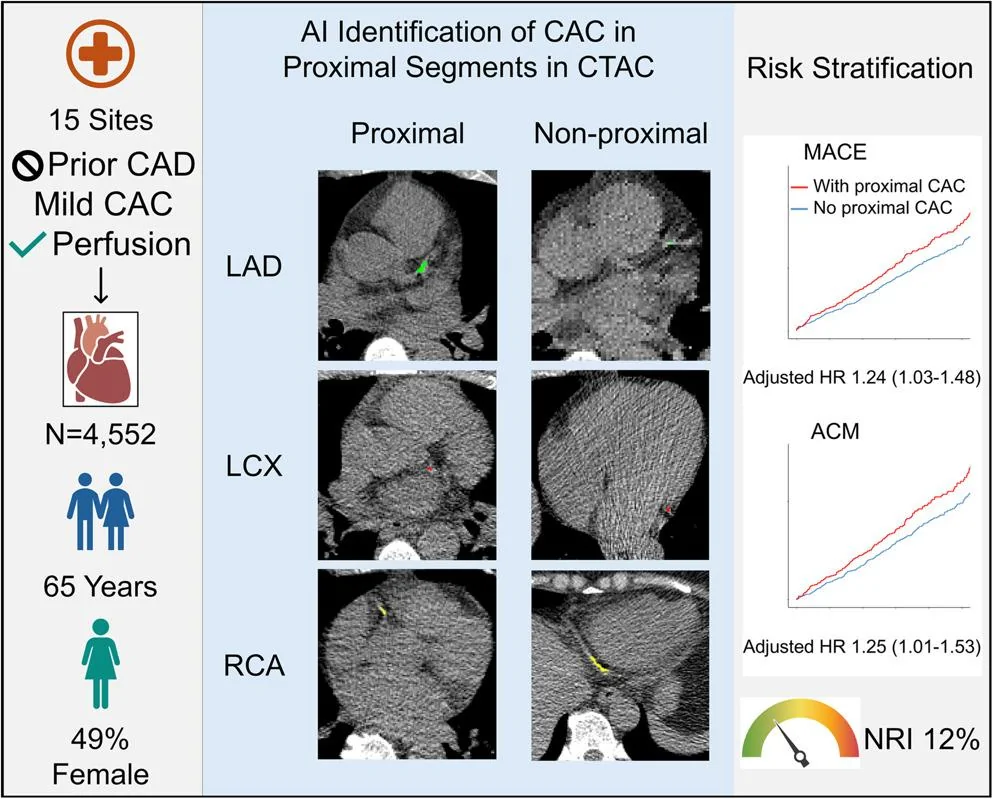

**Supplementary Information:**

The online version contains supplementary material available at https://doi.org/10.1007/s00259-026-08084-x.

## Introduction

Coronary artery calcium (CAC) evaluation is a critical anatomic tool for the assessment of CAD that can benefit patients undergoing hybrid myocardial perfusion imaging (**MPI**) [1], including single-photon emission computed tomography (**SPECT**) [2, 3] and positron emission tomography (**PET**) [4, 5]. CAC assessment can improve the detection of obstructive coronary artery disease (**CAD**) [6–9]. Several large studies have demonstrated that the CAC score is an independent and complementary predictor of cardiovascular risk in patients undergoing MPI [10–17].

Beyond the CAC score as a single measure, CAC location in the arteries may further improve the diagnostic and prognostic value of CAC phenotyping for MPI patients [18]. In particular, proximal plaques are more prone to rupture, leading to coronary thrombosis [19, 20]. As a result, the presence of proximal coronary plaque is associated with a higher cardiovascular risk in patients undergoing coronary computed tomography (**CT**) angiography [21, 22] or CAC CT scans [23]. In asymptomatic adults with mild CAC (CAC score 1–99 Agatston units [AU]), proximal CAC showed independent prognostic value in predicting MACE [23]. However, it is unknown whether the CAC location derived from low-dose, ungated CT attenuation correction (**CTAC**) scans (routinely acquired with hybrid imaging) provides independent prognostic value in a similar patient group [10, 12, 24]. Artificial intelligence (**AI**) has been applied to CTAC for CAC scoring [16, 25–28] and integrated risk prediction [14, 15, 29]. However, on these ultra-low-dose images, it can be difficult to accurately identify all CAC plaques or determine their exact location.

Previously, we developed a novel AI algorithm that integrates CAC and cardiac tissue segmentation to automatically classify CAC lesions into proximal and non-proximal categories [30]. In this study, we evaluated the prognostic significance of CAC proximal involvement for MPI patients with mild CAC and normal perfusion – the group who may benefit most from further risk stratification by proximal CAC –- in two large international multicenter registries comprising 15 sites.

## Materials and methods

### Study population

Consecutive patients with CTAC scans from hybrid SPECT/CT imaging (4 sites, *N* = 10,989) and PET/CT imaging (13 sites, *N* = 32,110; together 15 sites, 2 sites in both registries, *N* = 43,099) of two international multi-center registries [31–33] were studied. The registry protocols were approved by the Institutional Review Board at all participating sites, and sites either obtained written informed consent or a waiver of consent for the use of the deidentified data. The overall registries were approved by the investigational review board at Cedars-Sinai Medical Center and complied with the Declaration of Helsinki. Clinical variables collected at each site were de-identified and anonymized prior to transfer to the core site using dedicated software compliant with the Health Insurance Portability and Accountability Act (HIPAA). Patients with a history of coronary artery disease (defined as previous myocardial infarction, percutaneous coronary intervention, or coronary artery bypass grafts; *N* = 13,364) were excluded. Segmentation failure occurred in 63 patients (0.2% of 29,735) who were excluded. After exclusions, we had 29,672 patients.

### CT acquisition

All CT scans were acquired without contrast enhancement or cardiac gating. Details of acquisition protocols are shown in Supplement Table 1 for SPECT/CT and Supplement Table 2 for PET/CT.

### Perfusion imaging quantification

Stress total perfusion deficit (**TPD**) was used to determine perfusion status. Patients with a stress TPD < 5% were considered normal, and those with a value ≥ 5% were considered abnormal [34, 35]. Stress TPD was quantified using Quantitative Perfusion SPECT (QPS)/Quantitative Gated SPECT (QGS) software (Cedars-Sinai Medical Center, Los Angeles, California) [35, 36] for SPECT and the standard clinical quantitative PET package QPET (Cedars-Sinai Medical Center, Los Angeles, California) [37, 38] for PET. Myocardial flow reserve (**MFR**) was also quantified in PET patients, and an MFR > 2 was considered normal.

### Automated coronary artery calcium segmentation

A previously validated deep-learning model was used to segment vessel-specific CAC lesions (left anterior descending [**LAD**], left circumflex [**LCX**], and right coronary arteries [**RCA**]) [27, 39–41]. Lesions in the left main artery (LM) were included in LAD, following the development method of the segmentation model [39] and prior analyses of CAC spatial distribution [42]. Total CAC scores were determined from the CAC segmentation on CTAC scans using the Agatston method [43] and the standard threshold of 130 Hounsfield Units [27, 39, 44]. The scores of 1–99 AU were categorized as mild. CAC density was computed and adjusted to slice thickness [45].

### Identification of proximal CAC lesions

A previously reported AI algorithm [30] was used to identify CAC lesions in the proximal coronary segments. The algorithm used cardiac tissue segmentations [46] and analyzed distances between each CAC lesion from the previous step and a reference point automatically placed on the ascending aorta. On the slice of the CT scan showing the base of the left ventricle, the pixel in the ascending aorta with the shortest distance to the left ventricle was set as the reference point. The reference point was set as a proxy for the left ostium. In short, proximal CAC detection was performed by XGBoost (eXtreme Gradient Boosting) [47] integrating: (i) distance from the lesion to the reference point; (ii) location of the lesion defined as the center of mass in absolute image coordinates (coronal, sagittal, axial axes); (iii) location of the reference point in absolute image coordinates; (iv) the coronary artery having the lesion (LAD, LCX, or RCA). The algorithm was trained on dedicated CAC CT scans of 303 asymptomatic adults. Proximal location of CAC in each coronary artery was determined based on the branches (LAD based on the first diagonal branch, LCX based on the first obtuse marginal branches, RCA based on the distance from the ostium to the origin of the first acute marginal artery [23, 48]. Proximal segments were defined as proximal LAD (including LM), proximal LCX, and proximal RCA. The algorithm was adapted to CTAC by replacing image coordinates with physical distances from the reference point to the lesion along the three imaging axes.

### Clinical outcomes

The primary endpoint was major adverse cardiovascular events (**MACE**), defined as all-cause mortality (**ACM**), revascularization (percutaneous coronary intervention or coronary artery bypass grafting), admission for unstable angina, or non-fatal myocardial infarction. The secondary endpoint was all-cause mortality. In both SPECT and PET populations, follow-up for MACE was performed locally at each participating center [32, 33]. Non-fatal myocardial infarction and unstable angina are defined based on hospital admission for recent onset chest pain and admission diagnostics and myocardial infarction by elevated cardiac enzyme levels and ischemic ECG changes. All-cause mortality determination methods differ by country. In the United States, all-cause mortality is retrieved from the Social Security Death Index and is combined with MACE obtained from hospital electronic medical records or patient contact. In Canada, all-cause mortality is determined by chart review at the institute and physician’s office. In Israel, all-cause mortality is determined by the Ministry of Health database. In Mexico, all-cause mortality was obtained by chart review at the site or from patient contact. In Europe, MACE was obtained from the hospital electronic medical records. Revascularization details and data regarding hospitalization due to unstable angina or myocardial infarction were collected from email questionnaires, telephone contact, or medical records. For each patient considered to have a MACE, medical records regarding non-fatal events were reviewed and verified for correctness by site physicians. The first event for each patient was used as the MACE outcome.

### Statistics

Statistical analyses were performed using R (Version 4.4.1, R Foundation for Statistical Computing, Vienna, Austria) and Python 3.7.16. Continuous variables are presented as mean ± standard deviation for normally distributed data and median (interquartile interval [IQI]) for non-normally distributed data. A two-tailed P-value < 0.05 was considered statistically significant for the statistical tests mentioned above.

Cumulative event rates were computed with the Kaplan-Meier method. Survival curves were compared with the log-rank test. The multivariable Cox analysis included site, log(CAC + 1), CAC density, and clinical factors (age, sex, body mass index [**BMI**], hypertension, dyslipidemia, diabetes, family history of heart disease, and current smoker) as covariates, along with stress TPD. Site was included in the adjustment to account for underlying shared frailty in patient demographics, clinical profiles, and imaging parameters. We also performed vessel-specific analyses for the LAD, LCX, and RCA territories. In the PET sub-cohort, the risk of proximal CAC was assessed using the fully adjusted Cox model in the normal- and abnormal-MFR groups. The proportional hazard assumption was assessed with the Schoenfeld residual test. No violation was found. Hazard ratios (**HR**s) were presented with 95% confidence intervals (**CI**s).

To evaluate the incremental discriminative value of proximal CAC in such patient groups, the proximal involvement was added to a base Cox multivariable model as a nested model. The base model included scan sites, CAC score and density, clinical risk factors, and stress TPD. The prediction performance of the two models was assessed by Harrell’s C-index and compared using the likelihood ratio test. The incremental value was estimated by the continuous net reclassification index (**NRI**) [49].

## Results

### Patient characteristics

**Fig. 1:**
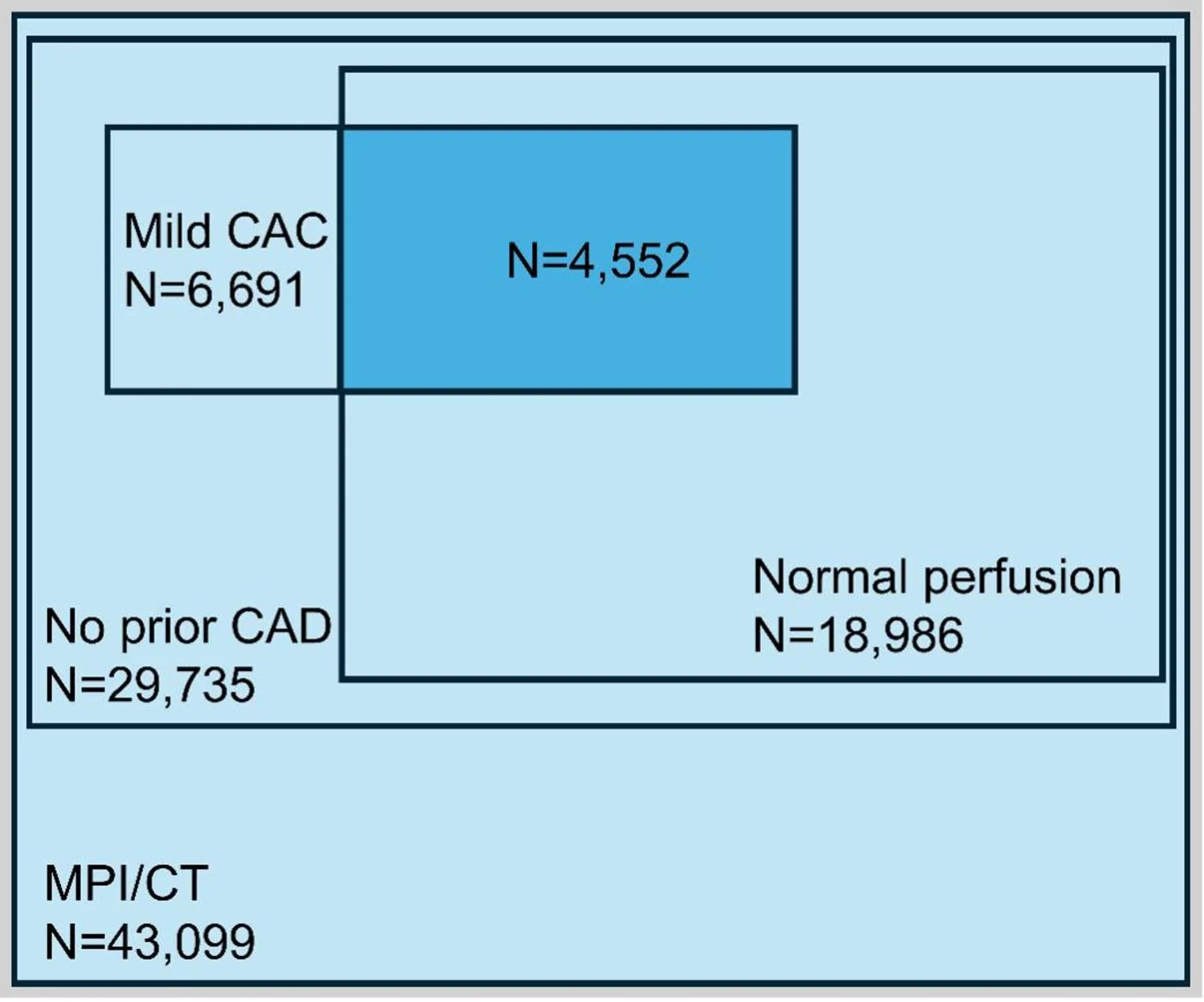
Study population. From hybrid myocardial perfusion imaging (MPI)/CT (N=43,099), the study sample (N=4,552) was selected for 1) no prior coronary artery disease (CAD), 2) mild coronary artery calcium (CAC), and 3) normal perfusion

In total, 18,986 (64% of 29,672) patients had normal perfusion (stress TPD < 5%). The deep learning model identified 17,830 (60% of 29,672) patients with CAC, of whom 6,691 had mild CAC (1–99). Together, we recognized 4,552 patients with mild CAC and normal perfusion for subsequent analyses (Fig. 1). Among these patients, the algorithm for proximal lesion detection determined 1,730 (38%) patients having proximal CAC. The baseline characteristics for patients with and without proximal CAC are summarized in Table 1. Patients with proximal CAC were significantly older (mean age 67 years versus 63 years), had a lower BMI (mean 29 kg/m^2^ versus 31 kg/m^2^), and were more likely to have dyslipidemia (64% versus 60%), but less likely to have diabetes (27% versus 30%). Patients with proximal CAC had higher total CAC scores (median 39 AU versus 22 AU), lower non-proximal CAC scores (median 0 versus 22 AU), and denser calcification (2.0 versus 1.8). No significant difference was found in sex, hypertension, family history of CAD, or stress TPD.

**Table 1:** Baseline demographic and clinical characteristics by CAC proximal involvement

	With proximal CAC	No proximal CAC	*P*-value
Number of patients	1,730	2,822	
Age, years	67 ± 11	63 ± 12	< 0.001
Male	886 (51%)	1,452 (51%)	0.9
Body mass index, kg/m^2^	29 ± 6	31 ± 7	< 0.001
Hypertension	1,212 (70%)	1,939 (69%)	0.3
Diabetes	459 (27%)	851 (30%)	0.009
Dyslipidemia	1,106 (64%)	1,687 (60%)	0.005
Family history of CAD	461 (27%)	708 (25%)	0.2
Current Smoker	413 (24%)	600 (21%)	0.040
MPI types			< 0.001
SPECT/CT	598 (35%)	763 (27%)	
PET/CT	1,132 (65%)	2,059 (73%)	
Stress TPD, %	1.6 (0.6, 2.9)	1.7 (0.7, 2.9)	0.4
CAC score, AU	39 (19, 65)	22 (11, 47)	< 0.001
Proximal CAC score, AU	23 (12, 43)	0	< 0.001
Non-proximal CAC score, AU	0 (0, 19)	22 (11, 47)	< 0.001
CAC density	2.0 (1.6, 2.4)	1.8 (1, 2)	< 0.001
Follow-up duration, years	3.5 (2.0, 5.1)	3.8 (2.1, 5.3)	0.001
MACE	251 (15%)	348 (12%)	0.035
All-cause mortality	185 (11%)	259 (9%)	0.094

Continuous variables are presented as mean ± standard deviation or median (lower quartile, upper quartile). Categorical variables are presented as counts (percentages). *AU* Agatston unit, *CAC* coronary artery calcium, *CAD* coronary artery disease, *MACE* major adverse cardiovascular events, *MPI* myocardial perfusion imaging, *PET* positron emission tomography, *SPECT* single-photon emission computed tomography, *TPD* total perfusion deficit

### Prognostic values of proximal involvement

**Fig. 2:**
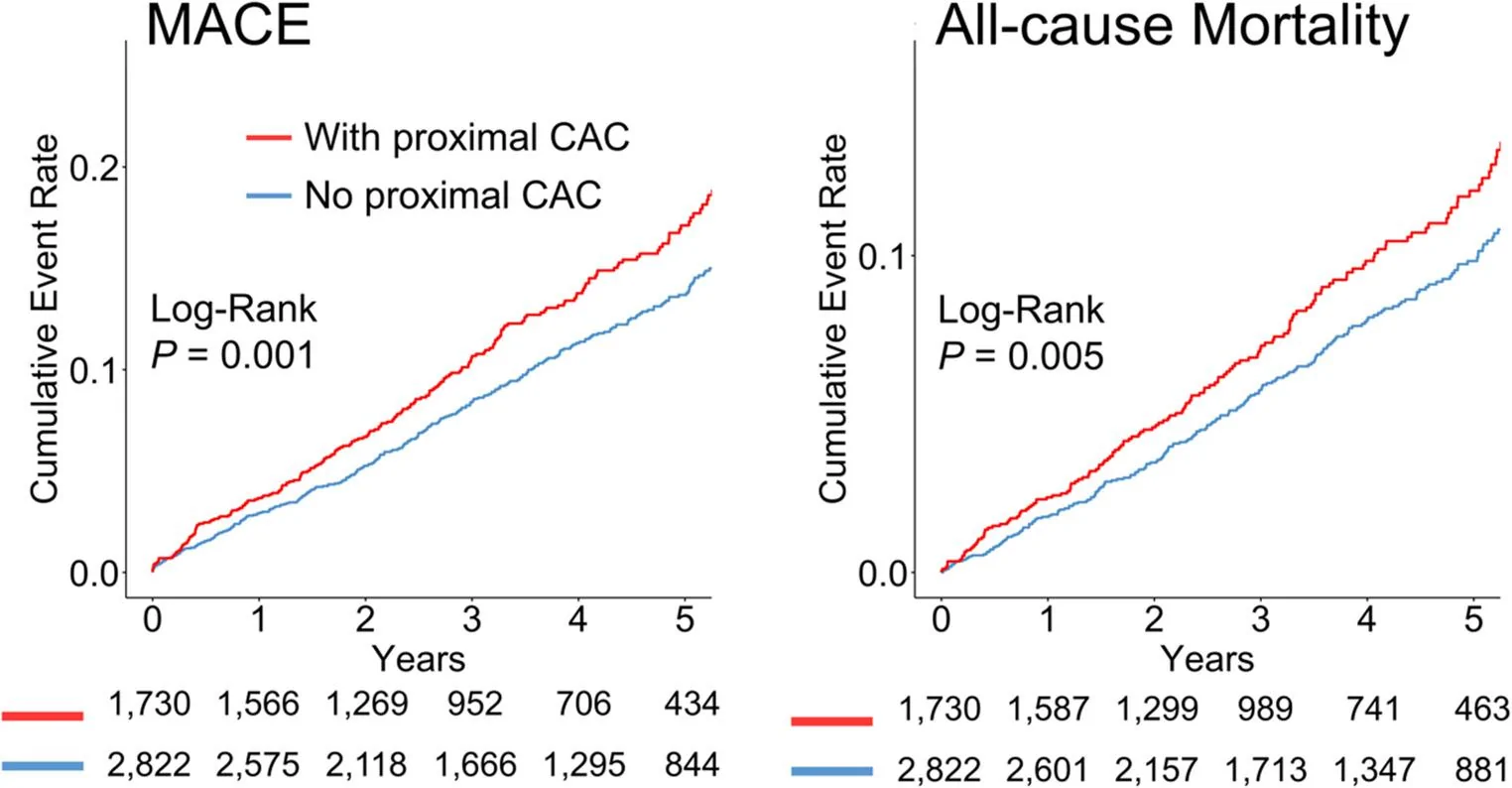
Kaplan-Meier survival curves stratified by the proximal involvement. Kaplan-Meier curves showing cumulative rates of major adverse cardiovascular events (MACE; left) and all-cause mortality (right). P-values from the log-rank test were shown

During the follow-up period of a median of 3.6 (IQI 2.1, 5.2) years, 599 (13%) patients had MACE, and 444 (10%) died. The risk stratification by proximal involvement is shown in Fig. 2. Patients with proximal CAC had significantly higher cumulative MACE (*P* = 0.001) and all-cause mortality (*P* = 0.005).

Proximal CAC was associated with an increased risk of MACE (adjusted HR 1.38, 95% CI 1.15–1.64, *P* < 0.001) and all-cause mortality (adjusted HR 1.44, 95% CI 1.18–1.77, *P* < 0.001) after adjusting for the total CAC score and imaging site (Table 2). Additional adjustment of CAC density gave the same adjusted HRs for both outcomes. After further adjustment of age, sex, BMI, and medical history, the increased risk of proximal CAC remained significant for MACE (adjusted HR 1.23, 95% CI 1.03–1.48, *P* = 0.020) and all-cause mortality (adjusted HR 1.24, 95% CI 1.01–1.53, *P* = 0.040). The significance was retained after accounting for stress TPD from perfusion imaging (MACE: adjusted HR 1.24, 95% CI 1.03–1.48, *P* = 0.020; all-cause mortality: adjusted HR 1.25, 95% CI 1.01–1.53, *P* = 0.036). Results of univariable and multivariable Cox regression analyses are provided in Supplement Fig. 1. Vessel-specific analyses showed that in patients with CAC only in the LCX territory, proximal involvement was associated with a 2.7 to 3.3-fold increase of risk (MACE: adjusted HR 3.29, 95% CI 1.94–5.58, *P* < 0.001; all-cause mortality: adjusted HR 2.69, 95% CI 1.42–5.09, *P* = 0.002; Supplement Table 3). In the PET sub-cohort (Supplement Table 4), the increased risk of all-cause mortality from proximal CAC was observed in the normal-MFR group (adjusted HR 1.46, 95% CI 1.09–1.95, *P* = 0.011; Supplement Table 5).

**Table 2:** Multivariable Cox regression of outcomes and CAC proximal involvement

Outcome	Model	Hazard ratio	95% CI	*P* value
MACE	Model 1	1.38	1.15–1.64	< 0.001
	Model 2	1.37	1.15–1.64	< 0.001
	Model 3	1.23	1.03–1.47	0.023
	Model 4	1.24	1.03–1.48	0.020
ACM	Model 1	1.44	1.18–1.77	< 0.001
	Model 2	1.45	1.18–1.78	< 0.001
	Model 3	1.24	1.01–1.53	0.040
	Model 4	1.25	1.01–1.53	0.036

Model 1: proximal involvement, total CAC score, site

Model 2: Model 1 plus CAC density

Model 3: Model 2 plus age, sex, body mass index, hypertension, diabetes, dyslipidemia, family history of coronary heart disease, current smoker

Model 4: Model 3 plus stress total perfusion deficit

Adjusted hazard ratios with 95% confidence intervals (CIs) are from Cox multivariable models. *ACM* all-cause mortality, *CAC* coronary artery calcium, *MACE* major adverse cardiovascular events

For the incremental prognostic value of proximal involvement, the base model reached a C-index of 0.645 ± 0.013 for the association with MACE (Supplement Table 6). The nested model, including the proximal involvement, increased the C-index to 0.648 ± 0.013 (*P* = 0.020). In the continuous NRI analysis over a 4-year interval, proximal CAC measurements resulted in an overall improvement of 12% (95% CI 2%-19%) of MACE cases. For all-cause mortality, proximal involvement improved the C-index from 0.680 ± 0.015 to 0.683 ± 0.014 (*P* = 0.037) with an overall NRI of 12% (95% CI 1%-24%).

**Fig. 3:**
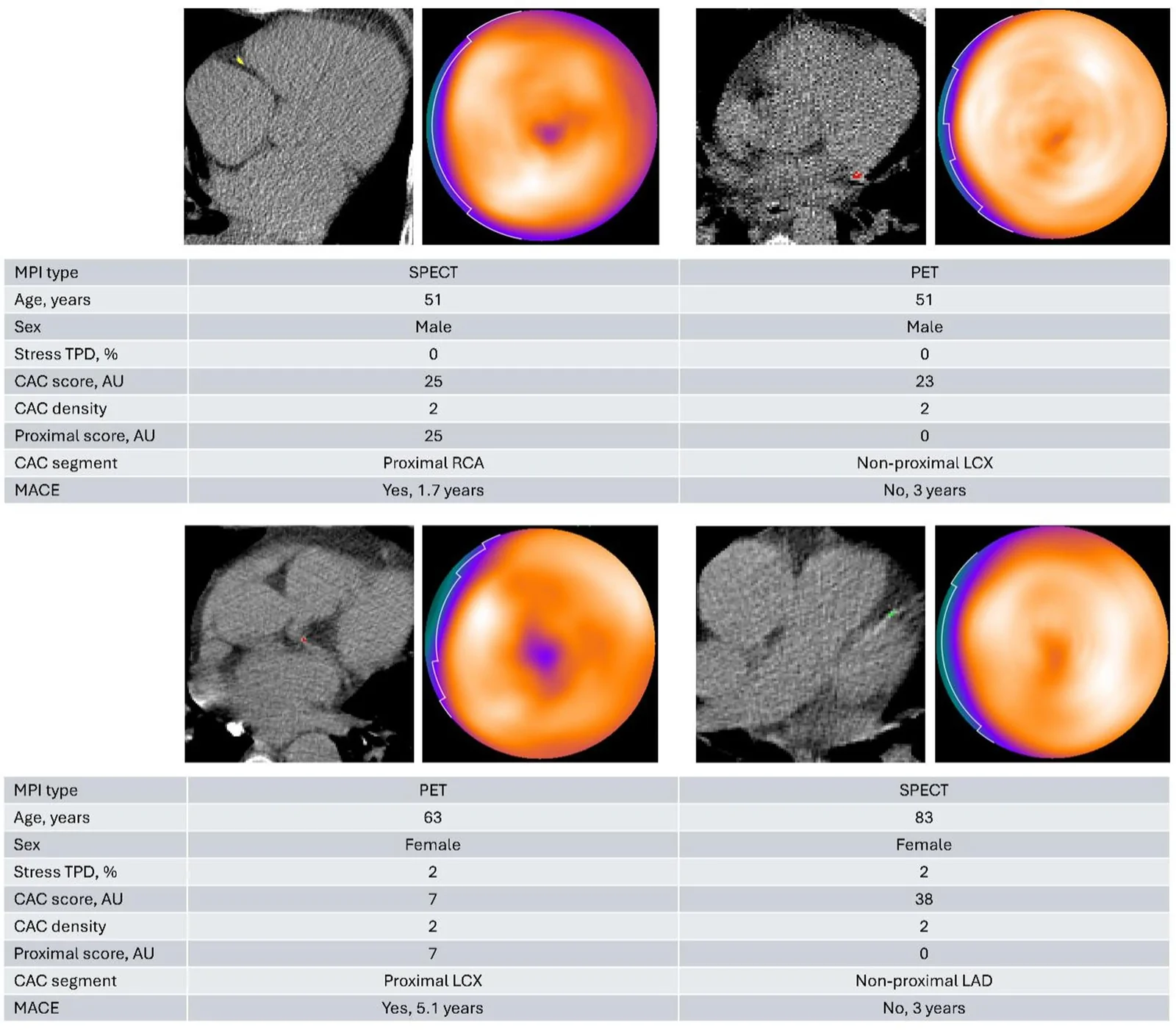
Illustration of proximal involvement and outcome. Two pairs of patient cases are presented. For each case, the CAC lesion is shown on the left in ungated non-contrast CT, and the stress perfusion polar map is shown on Accepted manuscript the right. The lesion in the left anterior descending artery (LAD) is shown in green, the left circumflex artery (LCX) lesions in red, and the right coronary artery (RCA) lesion in yellow. AU, Agatston Unit; CAC, coronary artery calcium; MACE, major adverse cardiovascular event; MPI, myocardial perfusion imaging; TPD, total perfusion deficit

The top panel of case illustrations (Fig. 3) shows two male patients in their 50s with similar CAC scores and densities, and stress TPD both as 0. The patient with CAC in the proximal RCA had MACE 1.7 years after the SPECT scan, whereas the patient with CAC in the non-proximal LCX remained free of MACE (3-year follow-up). The bottom panel shows two female patients. The patient with CAC in the proximal LCX was younger and had a lower CAC score. She had MACE 5.1 years after the PET scan. The older patient with CAC in the non-proximal LAD remained free of MACE (3 years).

## Discussion

In this international multi-center study, we identified CAC in proximal coronary segments from low-dose non-contrast ungated CTAC scans using an AI approach and then evaluated its prognostic importance. We demonstrated the feasibility of a fully automated pipeline for segmenting total CAC and identifying proximal CAC on CTAC scans. We found 9,961 (56%) patients with CAC in proximal coronary segments among 17,830 patients with CAC. In patients with mild CAC scores and normal perfusion, proximal CAC was independently associated with an increased risk of MACE and ACM. In patients with normal flow reserve, the independent increase in risk of ACM remained significant. Proximal CAC improved risk stratification beyond CAC score and density and other clinical risk factors, providing risk reclassification, particularly for patients without proximal CAC. Our results suggest that AI-enabled identification of proximal CAC could provide additional prognostic value for this patient group.

This is the first study to identify CAC in proximal coronary segments from low-dose non-contrast ungated CTAC scans and to evaluate the prognostic utility of proximal calcification in patients with suspected CAD, specifically those with mild CAC and normal perfusion. In this group from our international cohort, 37% of the patients had proximal CAC identified by AI. These patients were significantly older, more likely to have dyslipidemia, but were less likely to have higher BMI and diabetes. As in previous studies [23, 30], proximal CAC stratified the risk for MACE in the mild-CAC population. We also showed that proximal CAC had a similar stratification power for ACM risk. Patients with proximal CAC had higher cumulative event rates of MACE and ACM than those without proximal CAC. In addition, we found that in this group, patients with proximal CAC had significantly higher total CAC scores and density. After adjusting for CAC score and density [45, 50], the multivariable Cox regression models showed that proximal involvement was associated with a 1.3-fold increase in MACE risk and a 1.4-fold increase in ACM risk. The hazard ratios remained significant after additional adjustment for demographic information, clinical risk factors, and stress perfusion deficit. These suggest that proximal CAC is an independent and complementary predictor for MACE and ACM in this population.

An important implication from our study was that the absence of proximal CAC may help identify a lower-risk subgroup among patients with normal perfusion and mild CAC. This patient population represents a unique challenge for clinical management. Abnormal perfusion is frequently used to guide revascularization decisions and may help target medical therapies for CAD [51]. With respect to initiating medical therapy, statin therapy is generally considered indicated in patients with CAC > 100 [52]. However, among patients with CAC 1-100, the need for this therapy is less clear, with a number needed to treat of 100, compared with 12 among patients with CAC > 100 [53]. In addition, CAC 1–99 showed no prognostic difference for ACM compared to CAC = 0 in patients who underwent PET scans, particularly in the normal perfusion group [4]. Assuming that the risk of MACE and ACM is proportional to the benefit of statin therapy, the presence or absence of proximal CAC in patients with mild CAC scores may help identify patients more likely to benefit from preventive cardiovascular therapies.

We applied our fully automated pipeline to quantify proximal CAC from CTAC scans without comparing its accuracy against expert annotations. AI identification and measurement may differ from clinicians’ readings. However, the pipeline achieved high agreement with experts in the training dataset during the algorithm development. The deep-learning CAC segmentation combined LM lesions into the LAD territory due to the challenge inherent in separating these two territories and in assessing proximal versus distal segmentation of the LM. Future studies could evaluate the independent value of LM calcification segmentation. Vessel-specific analyses would also benefit from LM CAC segmentation and larger samples of vessel-specific disease. Additionally, classification of proximal CAC from CTAC is not routinely applied, and therefore, there is no clear ground truth. The average follow-up period was relatively short, which might not be sufficiently long to fully capture the long-term prognostic implications. We used stress TPD, a quantitative measure, to define normal perfusion. Although stress TPD is a well-validated quantitative measure of overall perfusion abnormality, future studies could evaluate the utility of these measures in patients with normal perfusion as defined by expert assessment using summed stress or summed difference scores. This approach could potentially be applied to other CT scans, such as coronary CT angiography. Dedicated studies would be needed to determine the incremental benefit of proximal CAC compared to standard reporting or segmental involvement scores in those scans. The use of multi-center scans with multiple imaging protocols makes results more robust and generalizable but may limit precision in estimating associated risk. Our cohort was at a high risk of MACE compared to healthy cohorts, which may be a reflection of the high prevalence of comorbidities such as diabetes and hypertension or selection bias related to the need for testing to evaluate for CAD. We focused on the prognostic value of proximal CAC in patients with suspected CAD and mild CAC because prior studies in asymptomatic adults suggested that mild CAC may provide risk stratification beyond the absence of CAC. Whether this prognostic value extends to symptomatic patients with suspected CAD remains unclear. Although the association between proximal CAC and outcomes remained significant in the fully adjusted models, the effect estimates were modest, and the confidence intervals were close to unity. Therefore, the findings should be interpreted as evidence of a relatively small but potentially clinically relevant independent prognostic association. Lastly, the retrospective design of the two registries limits our findings to correlation rather than causation, and future prospective studies are warranted.

## Conclusion

Proximal CAC on CT attenuation maps can be identified using an AI pipeline and is associated with MACE and ACM in patients with suspected CAD, specifically those with mild CAC and normal perfusion. The identification may improve risk stratification for these patients.

## Supplementary Information

Below is the link to the electronic supplementary material.

Supplementary Material 1 (DOCX 239 KB)

## Data Availability

To the extent allowed by data sharing agreements and IRB protocols, data from this manuscript will be shared upon written request.

## Abbreviations

ACM: all-cause mortality
AI: artificial intelligence
CAC: coronary artery calcium
CTAC: computed tomography for attenuation correction
MACE: major adverse cardiovascular event
MPI: myocardial perfusion imaging
PET: positron emission tomography
SPECT: single-photon emission computed tomography

## References

[1] El Yaman A, Alwan M, El Ghazawi A, Al-Mallah MH, Al Rifai M. Advances in nuclear hybrid imaging: The role of SPECT/CT, PET/CT, and PET/MRI in cardiovascular diseases. Prog Cardiovasc Dis. 2025;93:43–50. 10.1016/j.pcad.2025.09.007. 40962131

[2] Hijazi W, Feng Y, Southern DA, Chew D, Filipchuk N, Har B, et al. Impact of myocardial perfusion and coronary calcium on medical management for coronary artery disease. Eur Heart J Cardiovasc Imaging. 2024;25:482–90. 10.1093/ehjci/jead288. PMC10966327 37889992

[3] Engbers EM, Timmer JR, Mouden M, Knollema S, Jager PL, Ottervanger JP. Changes in cardiovascular medication after coronary artery calcium scanning and normal single photon emission computed tomography myocardial perfusion imaging in symptomatic patients. Am Heart J. 2017;186:56–62. 10.1016/j.ahj.2017.01.009. 28454833

[4] Patel KK, Peri-Okonny PA, Qarajeh R, Patel FS, Sperry BW, McGhie AI, et al. Prognostic Relationship Between Coronary Artery Calcium Score, Perfusion Defects, and Myocardial Blood Flow Reserve in Patients With Suspected Coronary Artery Disease. Circ Cardiovasc Imaging. 2022;15:e012599. 10.1161/CIRCIMAGING.121.012599. PMC9018603 35414185

[5] Mannarino T, D’Antonio A, Assante R, Zampella E, Gaudieri V, Petretta M, et al. Combined evaluation of CAC score and myocardial perfusion imaging in patients at risk of cardiovascular disease: where are we and what do the data say. J Nucl Cardiol. 2023;30:2349–60. 10.1007/s12350-023-03288-2. PMC10682302 37162738

[6] Schepis T, Gaemperli O, Koepfli P, Namdar M, Valenta I, Scheffel H, et al. Added value of coronary artery calcium score as an adjunct to gated SPECT for the evaluation of coronary artery disease in an intermediate-risk population. J Nucl Med. 2007;48:1424–30. 10.2967/jnumed.107.040758. 17785727

[7] Berman DS, Wong ND, Gransar H, Miranda-Peats R, Dahlbeck J, Hayes SW, et al. Relationship between stress-induced myocardial ischemia and atherosclerosis measured by coronary calcium tomography. J Am Coll Cardiol. 2004;44:923–30. 10.1016/j.jacc.2004.06.042. 15312881

[8] Miller RJH, Gransar H, Rozanski A, Dey D, Al-Mallah M, Chow BJW, et al. Simplified Approach to Predicting Obstructive Coronary Disease With Integration of Coronary Calcium: Development and External Validation. J Am Heart Assoc. 2023;12:e031601. 10.1161/jaha.123.031601. PMC10863788 38108259

[9] Brodov Y, Gransar H, Dey D, Shalev A, Germano G, Friedman JD, et al. Combined Quantitative Assessment of Myocardial Perfusion and Coronary Artery Calcium Score by Hybrid 82Rb PET/CT Improves Detection of Coronary Artery Disease. J Nucl Med. 2015;56:1345–50. 10.2967/jnumed.114.153429. 26159582

[10] Sharma V, Mughal L, Dimitropoulos G, Sheikh A, Griffin M, Moss A, et al. The additive prognostic value of coronary calcium score (CCS) to single photon emission computed tomography myocardial perfusion imaging (SPECT-MPI)-real world data from a single center. J Nucl Cardiol. 2021;28:2086–96. 10.1007/s12350-019-01965-9. 31797319

[11] Trpkov C, Savtchenko A, Liang Z, Feng P, Southern DA, Wilton SB, et al. Visually estimated coronary artery calcium score improves SPECT-MPI risk stratification. Int J Cardiol Heart Vasc. 2021;35:100827. 10.1016/j.ijcha.2021.100827. PMC8233133 34195354

[12] Engbers EM, Timmer JR, Ottervanger JP, Mouden M, Knollema S, Jager PL. Prognostic value of coronary artery calcium scoring in addition to single-photon emission computed tomographic myocardial perfusion imaging in symptomatic patients. Circ Cardiovasc Imaging. 2016;9. 10.1161/CIRCIMAGING.115.003966. 27165700

[13] Chang SM, Nabi F, Xu J, Pratt CM, Mahmarian AC, Frias ME, et al. Value of CACS compared with ETT and myocardial perfusion imaging for predicting long-term cardiac outcome in asymptomatic and symptomatic patients at low risk for coronary disease: clinical implications in a multimodality imaging world. JACC Cardiovasc Imaging. 2015;8:134–44. 10.1016/j.jcmg.2014.11.008. 25677886

[14] Feher A, Pieszko K, Miller R, Lemley M, Shanbhag A, Huang C, et al. Integration of coronary artery calcium scoring from CT attenuation scans by machine learning improves prediction of adverse cardiovascular events in patients undergoing SPECT/CT myocardial perfusion imaging. J Nucl Cardiol. 2023;30:590–603. 10.1007/s12350-022-03099-x. PMC12969475 36195826

[15] Michalowska AM, Zhang W, Shanbhag A, Miller RJ, Lemley M, Ramirez G, et al. Holistic AI analysis of hybrid cardiac perfusion images for mortality prediction. medRxiv. 2024. 10.1101/2024.04.23.24305735. PMC11906890 40082599

[16] Miller RJH, Pieszko K, Shanbhag A, Feher A, Lemley M, Killekar A, et al. Deep Learning Coronary Artery Calcium Scores from SPECT/CT Attenuation Maps Improve Prediction of Major Adverse Cardiac Events. J Nucl Med. 2023;64:652–8. 10.2967/jnumed.122.264423. PMC10071789 36207138

[17] Miller RJH, Han D, Singh A, Pieszko K, Slomka PJ, Gransar H, et al. Relationship between ischaemia, coronary artery calcium scores, and major adverse cardiovascular events. Eur Heart J Cardiovasc Imaging. 2022;23:1423–33. 10.1093/ehjci/jeac082. 35608211

[18] Jiang Q, Liu B, Yang L, Wang Y, Yu W, Zhang F, et al. The value of regional and global CACS combined with SPECT MPI in detecting obstructive CAD: a retrospective real-world comparative study. BMC Cardiovasc Disord. 2023;23:12. 10.1186/s12872-023-03051-y. PMC9832744 36631747

[19] Cheruvu PK, Finn AV, Gardner C, Caplan J, Goldstein J, Stone GW, et al. Frequency and distribution of thin-cap fibroatheroma and ruptured plaques in human coronary arteries: a pathologic study. J Am Coll Cardiol. 2007;50:940–9. 10.1016/j.jacc.2007.04.086. 17765120

[20] Wang JC, Normand SL, Mauri L, Kuntz RE. Coronary artery spatial distribution of acute myocardial infarction occlusions. Circulation. 2004;110:278–84. 10.1161/01.Cir.0000135468.67850.F4. 15249505

[21] Han D, Chen B, Gransar H, Achenbach S, Al-Mallah MH, Budoff MJ, et al. Prognostic significance of plaque location in non-obstructive coronary artery disease: from the CONFIRM registry. Eur Heart J Cardiovasc Imaging. 2022;23:1240–7. 10.1093/ehjci/jeab223. 34791117

[22] Han D, Lin A, Kuronuma K, Tzolos E, Kwan AC, Klein E, et al. Association of Plaque Location and Vessel Geometry Determined by Coronary Computed Tomographic Angiography With Future Acute Coronary Syndrome-Causing Culprit Lesions. JAMA Cardiol. 2022;7:309–19. 10.1001/jamacardio.2021.5705. PMC8792800 35080587

[23] Tummala R, Han D, Friedman J, Hayes S, Thomson L, Gransar H, et al. Association between plaque localization in proximal coronary segments and MACE outcomes in patients with mild CAC: Results from the EISNER study. Am J Prev Cardiol. 2022;12:100423. 10.1016/j.ajpc.2022.100423. PMC9529495 36199447

[24] Chang SM, Nabi F, Xu J, Peterson LE, Achari A, Pratt CM, et al. The coronary artery calcium score and stress myocardial perfusion imaging provide independent and complementary prediction of cardiac risk. J Am Coll Cardiol. 2009;54:1872–82. 10.1016/j.jacc.2009.05.071. 19892239

[25] van Velzen SGM, Lessmann N, Velthuis BK, Bank IEM, van den Bongard D, Leiner T, et al. Deep Learning for Automatic Calcium Scoring in CT: Validation Using Multiple Cardiac CT and Chest CT Protocols. Radiology. 2020;295:66–79. 10.1148/radiol.2020191621. PMC7106943 32043947

[26] van Velzen SGM, Dobrolinska MM, Knaapen P, van Herten RLM, Jukema R, Danad I, et al. Automated cardiovascular risk categorization through AI-driven coronary calcium quantification in cardiac PET acquired attenuation correction CT. J Nucl Cardiol. 2023;30:955–69. 10.1007/s12350-022-03047-9. PMC10261233 35851642

[27] Pieszko K, Shanbhag A, Killekar A, Miller RJH, Lemley M, Otaki Y, et al. Deep Learning of Coronary Calcium Scores From PET/CT Attenuation Maps Accurately Predicts Adverse Cardiovascular Events. Jacc-Cardiovascular Imaging. 2023;16:675–87. 10.1016/j.jcmg.2022.06.006. PMC13016421 36284402

[28] Miller RJH, Chareonthaitawee P, Slomka PJ. Artificial intelligence in nuclear cardiology: Enhancing diagnostic accuracy and efficiency. Prog Cardiovasc Dis. 2025;93:85–91. 10.1016/j.pcad.2025.09.010. 41038418

[29] Dekker M, Waissi F, Bank IEM, Isgum I, Scholtens AM, Velthuis BK, et al. The prognostic value of automated coronary calcium derived by a deep learning approach on non-ECG gated CT images from (82)Rb-PET/CT myocardial perfusion imaging. Int J Cardiol. 2021;329:9–15. 10.1016/j.ijcard.2020.12.079. 33412176

[30] Zhou J, Shanbhag AD, Han D, Michalowska AM, Buchwald M, Miller RJH, et al. Automated proximal coronary artery calcium identification using artificial intelligence: advancing cardiovascular risk assessment. Eur Heart J Cardiovasc Imaging. 2025. 10.1093/ehjci/jeaf007. PMC11879235 39821011

[31] Slomka PJ, Betancur J, Liang JX, Otaki Y, Hu LH, Sharir T, et al. Rationale and design of the REgistry of Fast Myocardial Perfusion Imaging with NExt generation SPECT (REFINE SPECT). J Nucl Cardiol. 2020;27:1010–21. 10.1007/s12350-018-1326-4. PMC6301135 29923104

[32] Miller RJH, Lemley M, Shanbhag A, Ramirez G, Liang JX, Builoff V, et al. The Updated Registry of Fast Myocardial Perfusion Imaging with Next-Generation SPECT (REFINE SPECT 2.0). J Nucl Med. 2024. 10.2967/jnumed.124.268292. PMC11533915 39362762

[33] Ramirez G, Lemley M, Shanbhag A, Kwiecinski J, Miller RJH, Kavanagh PB, et al. The REgistry of flow and perfusion imaging for artificial INtelligEnce with PET(REFINE PET): rationale and design. J Nucl Cardiol. 2025;102449. 10.1016/j.nuclcard.2025.102449. PMC13175774 40774620

[34] Berman DS, Kang X, Gransar H, Gerlach J, Friedman JD, Hayes SW, et al. Quantitative assessment of myocardial perfusion abnormality on SPECT myocardial perfusion imaging is more reproducible than expert visual analysis. J Nucl Cardiol. 2009;16:45–53. 10.1007/s12350-008-9018-0. PMC3569514 19152128

[35] Slomka PJ, Nishina H, Berman DS, Akincioglu C, Abidov A, Friedman JD, et al. Automated quantification of myocardial perfusion SPECT using simplified normal limits. J Nucl Cardiol. 2005;12:66–77. 10.1016/j.nuclcard.2004.10.006. 15682367

[36] Nakazato R, Tamarappoo BK, Kang X, Wolak A, Kite F, Hayes SW, et al. Quantitative upright-supine high-speed SPECT myocardial perfusion imaging for detection of coronary artery disease: correlation with invasive coronary angiography. J Nucl Med. 2010;51:1724–31. 10.2967/jnumed.110.078782. PMC3130607 20956478

[37] Dekemp RA, Declerck J, Klein R, Pan XB, Nakazato R, Tonge C, et al. Multisoftware reproducibility study of stress and rest myocardial blood flow assessed with 3D dynamic PET/CT and a 1-tissue-compartment model of 82Rb kinetics. J Nucl Med. 2013;54:571–7. 10.2967/jnumed.112.112219. 23447656

[38] Slomka PJ, Alexanderson E, Jácome R, Jiménez M, Romero E, Meave A, et al. Comparison of clinical tools for measurements of regional stress and rest myocardial blood flow assessed with 13 N-ammonia PET/CT. J Nucl Med. 2012;53:171–81. 10.2967/jnumed.111.095398. 22228795

[39] Williams MC, Shanbhag AD, Zhou J, Michalowska AM, Lemley M, Miller RJ, et al. Automated vessel specific coronary artery calcification quantification with deep learning in a large multi-center registry. Eur Heart J Cardiovasc Imaging. 2024. 10.1093/ehjci/jeae045. PMC11210989 38376471

[40] Pieszko K, Shanbhag A, Killekar A, Lemley M, Otaki Y, Van Kriekinge S, et al. Calcium scoring in low-dose ungated chest CT scans using convolutional long. -short term memory networks: SPIE; 2022. 10.1117/12.2613147 PMC9585987 36277935

[41] Shanbhag AD, Pieszko K, Miller RJH, Killekar A, Parker W, Gransar H, et al. Comparative analysis between convolutional long short-term memory networks and vision transformers for coronary calcium scoring in non. -contrast CT: SPIE; 2023.

[42] Brown ER, Kronmal RA, Bluemke DA, Guerci AD, Carr JJ, Goldin J, et al. Coronary calcium coverage score: determination, correlates, and predictive accuracy in the Multi-Ethnic Study of Atherosclerosis. Radiology. 2008;247:669–75. 10.1148/radiol.2473071469. PMC5410932 18413889

[43] Agatston AS, Janowitz WR, Hildner FJ, Zusmer NR, Viamonte M Jr., Detrano R. Quantification of coronary artery calcium using ultrafast computed tomography. J Am Coll Cardiol. 1990;15:827–32. 10.1016/0735-1097(90)90282-t. 2407762

[44] Robert JHM, Konrad P, Aakash S, Attila F, Mark L, Aditya K, et al. Deep Learning Coronary Artery Calcium Scores from SPECT/CT Attenuation Maps Improve Prediction of Major Adverse Cardiac Events. J Nucl Med. 2023;64:652. 10.2967/jnumed.122.264423. PMC10071789 36207138

[45] Criqui MH, Denenberg JO, Ix JH, McClelland RL, Wassel CL, Rifkin DE, et al. Calcium density of coronary artery plaque and risk of incident cardiovascular events. JAMA. 2014;311:271–8. 10.1001/jama.2013.282535. PMC4091626 24247483

[46] Wasserthal J, Breit HC, Meyer MT, Pradella M, Hinck D, Sauter AW, et al. TotalSegmentator: Robust Segmentation of 104 Anatomic Structures in CT Images. Radiol Artif Intell. 2023;5:e230024. 10.1148/ryai.230024. PMC10546353 37795137

[47] Chen T, Guestrin C. XGBoost: a scalable tree boosting system. Proc 22nd ACM SIGKDD Int Conf Knowl Discovery Data Min. 2016;785–94. 10.1145/2939672.2939785.

[48] Leipsic J, Abbara S, Achenbach S, Cury R, Earls JP, Mancini GJ, et al. SCCT guidelines for the interpretation and reporting of coronary CT angiography: a report of the Society of Cardiovascular Computed Tomography Guidelines Committee. J Cardiovasc Comput Tomogr. 2014;8:342–58. 10.1016/j.jcct.2014.07.003. 25301040

[49] Pencina MJ, D’Agostino RB, Sr., D’Agostino RB Jr., Vasan RS. Evaluating the added predictive ability of a new marker: from area under the ROC curve to reclassification and beyond. Stat Med. 2008;27:157–72. 10.1002/sim.2929. discussion 207 – 12. 17569110

[50] Criqui MH, Knox JB, Denenberg JO, Forbang NI, McClelland RL, Novotny TE, et al. Coronary Artery Calcium Volume and Density: Potential Interactions and Overall Predictive Value: The Multi-Ethnic Study of Atherosclerosis. JACC Cardiovasc Imaging. 2017;10:845–54. 10.1016/j.jcmg.2017.04.018. 28797404

[51] Hijazi W, Feng Y, Southern DA, Chew D, Filipchuk N, Mylonas I, et al. Association of medical therapies with survival according to SPECT MPI findings. J Nucl Cardiol. 2025;49:102229. 10.1016/j.nuclcard.2025.102229. 40324562

[52] Arnett DK, Blumenthal RS, Albert MA, Buroker AB, Goldberger ZD, Hahn EJ, et al. 2019 ACC/AHA Guideline on the Primary Prevention of Cardiovascular Disease: A Report of the American College of Cardiology/American Heart Association Task Force on Clinical Practice Guidelines. J Am Coll Cardiol. 2019;74:e177–232. 10.1016/j.jacc.2019.03.010. PMC7685565 30894318

[53] Mitchell JD, Fergestrom N, Gage BF, Paisley R, Moon P, Novak E, et al. Impact of Statins on Cardiovascular Outcomes Following Coronary Artery Calcium Scoring. J Am Coll Cardiol. 2018;72:3233–42. 10.1016/j.jacc.2018.09.051. PMC6309473 30409567

