# Supplementary material for: Hidden risk in normal myocardial perfusion scans: AI-detected proximal coronary calcium on CT attenuation maps improves prognosis
Source: Eur J Nucl Med Mol Imaging. 2026 Jul 28;53(12):7610–20. doi: 10.1007/s00259-026-08084-x (PMC13492496; doi:10.1007/s00259-026-08084-x)
Supplement: Supplementary file 1 [file 259_2026_8084_MOESM1_ESM.docx]

**Supplemental Material**

Supplement Table 1. CT acquisition protocols with SPECT imaging

| **Site** | **Number of patients** | **Scanner system** | **Slice thickness, mm** | **Tube current, mA** | **Tube voltage, kVp** |
| --- | --- | --- | --- | --- | --- |
| University of Calgary | 2,912 | GE Discovery 570 | 5 | 20 | 120 |
| Columbia University | 1,878 | Philips Precedence 16P | 3 | 30 | 120 |
| Ottawa Heart Institute | 1,433 | Siemens Symbia Intevo | 5 | 20-30 | 130 |
| Yale University | 4,766 | GE Discovery 570 | 2.5 | 60 or 150, based on body-mass index | 120 |

Supplement Table 2. CT acquisition protocols with PET imaging

| **Site** | **Number of patients** | **Scanner system** | **Slice thickness, mm** | **Tube current, mA** | **Tube voltage, kVp** |
| --- | --- | --- | --- | --- | --- |
| Brigham and Women’s Hospital | 7,200 | GE Discovery MI  GE Discovery MI RX  GE Discovery MI STE | 2.5-5 | 10-26 | 120-140 |
| Cedars-Sinai Medical Center | 5,002 | Siemens Biograph 64 TruePoint  Siemens Biograph 128 Vision Edge  GE Discovery 710 | 3 | 11-13 | 100 |
| Columbia University Irving Medical Center | 1,922 | Siemens Biograph 64 mCT Flow | 3 | 75 | 120 |
| Houston Methodist Academic Institute | 1,876 | Siemens Biograph 600 Vision Edge | 3 | 20-50 | 100-120 |
| Intermountain Medical Center | 5,907 | Siemens Biograph 16 TruePoint  Siemens Biograph 20 mCT  Siemens Biograph 40 mCT | 2 | 15-38 | 120-130 |
| University of Kansas Medical Center | 589 | GE Discovery MI | 3.75 | 14-75 | 120 |
| Montefiore Medical Center | 1,014 | Philips Gemini TF TOF 16  Philips Gemini TF TOF 64 | 3 | 110-185 | 120 |
| Mayo Clinic | 1,989 | GE Discovery 710 | 3.75 | 17-77 | 120 |
| National Autonomous University of Mexico | 323 | Siemens Biograph 64 TruePoint  Siemens Biograph Vision 600 | 3 | 33-580 | 120 |
| University of Naples Federico II | 455 | Philips Ingenuity TF  GE Discovery Mi | 3 | 35-100 | 120-140 |
| Ottawa Heart Institute | 3,934 | GE Discovery 690  GE Discovery 600  Siemens Biograph 600 Vision Edge | 3-5 | 20-60 | 120 |
| West Los Angeles Veterans Affairs Medical Center | 1,150 | Siemens Biograph 64 mCT  Siemens Biograph 64 Vision 600 | 3 | 70-200 | 120 |
| University Hospital Zurich | 749 | GE Discovery STE  GE Discovery RX  GE Discovery LS  GE Discovery HR | 3.75-5 | 140 | 40-240 |

Supplement Table 3. Vessel-specific associations with outcomes.

| Vessel | Total | With proximal CAC | No proximal CAC | Outcome | Hazard ratio | 95% CI | P value |
| --- | --- | --- | --- | --- | --- | --- | --- |
| LAD | 1,484 | 823 | 661 | MACE | 1.25 | 0.92-1.70 | 0.15 |
|  |  |  |  | ACM | 1.28 | 0.89-1.83 | 0.2 |
| LCX | 820 | 114 | 706 | MACE | 3.29 | 1.94-5.58 | < 0.001 |
|  |  |  |  | ACM | 2.69 | 1.42-5.09 | 0.002 |
| RCA | 1054 | 201 | 853 | MACE | 1.44 | 0.94-2.20 | 0.090 |
|  |  |  |  | ACM | 1.43 | 0.89-2.32 | 0.14 |

The number of patients having CAC only in either LAD (together with LM), LCX, or RCA is presented with proximal involvement. Hazard ratios and 95% confidence intervals (CIs) were estimated after adjustment for total coronary artery calcium (CAC) score, site, CAC density, age, sex, body mass index, hypertension, diabetes, dyslipidemia, family history of coronary heart disease, current smoker, and stress total perfusion deficit. ACM, all-cause mortality; LAD, left anterior descending artery; LCX, left circumflex artery; LM, left main artery; MACE, major adverse cardiovascular events; RCA, right coronary artery.

Supplement Table 4. Myocardial blood flow and flow reserve in the PET sub-cohort, N=3191.

|  | With proximal CAC | No proximal CAC | P-value |
| --- | --- | --- | --- |
| Number of patients having PET/CT | 1132 | 2059 |  |
| Stress MBF | 2.6 (2.1, 3.1) | 2.6 (2.1, 3.1) | 0.5 |
| MFR | 2.5 (2.1, 3.0) | 2.5 (2.1, 3.1) | > 0.9 |

Stress myocardial blood flow (MBF) and myocardial flow reserve (MFR) are presented as median (lower quartile, upper quartile). CAC, coronary artery calcium.

Supplement Table 5. Multivariable Cox regression of outcome stratified by flow reserve.

| MFR | Outcome | Hazard ratio | 95% CI | P value |
| --- | --- | --- | --- | --- |
| Normal (N=2511) | MACE | 1.28 | 1.00-1.64 | 0.055 |
|  | ACM | 1.46 | 1.09-1.95 | 0.011 |
| Abnormal (N=680) | MACE | 1.09 | 0.77-1.53 | 0.6 |
|  | ACM | 1.01 | 0.69-1.47 | > 0.9 |

Hazard ratios and 95% confidence intervals (CIs) were estimated in the normal (MFR > 2) and abnormal (MFR ≤ 2) after adjustment for total coronary artery calcium (CAC) score, site, CAC density, age, sex, body mass index, hypertension, diabetes, dyslipidemia, family history of coronary heart disease, current smoker, and stress total perfusion deficit. ACM, all-cause mortality; MACE, major adverse cardiovascular events; MFR, myocardial flow reserve.
Supplement Table 6. Likelihood ratio test and continuous net reclassification analysis.

| Outcome | Base C-index | Nested C-index | Likelihood ratio test P-value | NRI (95% CI) |
| --- | --- | --- | --- | --- |
| MACE | 0.645 ± 0.013 | 0.648 ± 0.013 | 0.020 | 12% (2%-19%) |
| ACM | 0.680 ± 0.015 | 0.683 ± 0.014 | 0.037 | 12% (1%-24%) |

C-indices were shown with standard deviations. Base model: adjustment 4 in Table 2; nested model: base model + proximal involvement. ACM, all-cause mortality; CI, confidence interval; MACE, major adverse cardiovascular events; NRI, net reclassification index.

Supplement Figure 1. Associations with outcomes

Hazard ratios (HRs) with 95% confidence intervals (CIs) are shown for proximal CAC and confounding risk factors in univariable and multivariable Cox regressions. Site was included in the multivariable analyses, but the hazard ratios of different sites are not presented. BMI, body mass index; CAC, coronary artery calcium; CAD, coronary artery disease; MACE, major adverse cardiovascular event; TPD, total perfusion deficit.
